# Evaluation of Rice Quality Storage Stability: From Variety Screening to Trait Identification

**DOI:** 10.3390/plants14030356

**Published:** 2025-01-24

**Authors:** Jinyu Tian, Guangmei Ji, Jiafeng Zhang, Danqiu Luo, Fang Zhang, Lijiang Li, Mingjin Jiang, Dawei Zhu, Min Li

**Affiliations:** 1 Rice Research Institute of Guizhou Province, Guizhou Academy of Agricultural Sciences, Guiyang 550025, China; jinyu230811@163.com (J.T.); jiaguangmei2008@126.com (G.J.); 13688521449@136.com (J.Z.); ldq18198101384@163.com (D.L.); 15329577052@163.com (F.Z.); lljiang8421@163.com (L.L.); mj_jiang2008@163.com (M.J.); 2 Rice Product Quality Supervision and Inspection Center, Ministry of Agriculture and Rural Affairs, China National Rice Research Institute, Hangzhou 310006, China

**Keywords:** rice, grain quality, storage stability, variety screening, characterization analysis

## Abstract

Rice, a staple global food crop, requires the maintenance of its quality stability during storage. This study aimed to screen rice varieties with high storage stability and elucidate their traits. Thirty-four widely cultivated varieties were selected to examine the changes in grain quality after one-year natural storage. The normalization method, hierarchical analysis, and cluster analysis were used to identify rice varieties maintaining their grain quality during storage. Meanwhile, the yield and its components, panicle traits, grain size, grain major component content, physiological indicators (such as antioxidant enzyme activity), and key growth stages were analyzed at rice maturity. The results showed that the processing, appearance, and eating quality of rice declined after storage. Specifically, the chalkiness degree increased significantly by 32.4%, while the cooked rice appearance, texture, and taste quality decreased significantly by 18.7%, 19.1%, and 14.2%, respectively. The grain quality storage stability was evaluated using a hierarchical analysis method based on the storage stability scores of the brown rice rate, milled rice rate, head milled rice rate, chalkiness degree, cooked rice appearance, cooked rice texture, and cooked rice taste. A judgment matrix was established, determining their corresponding weights of 0.0149, 0.0369, 0.0910, 0.286, 0.060, 0.148, and 0.364, respectively. Based on cluster analysis and the normalization method, these varieties were classified into three categories: high storage stability, intermediate storage stability, and low storage stability, accounting for 26.5%, 52.9%, and 20.6%, respectively. Finally, nine rice varieties with high storage stability were screened. The validation results of the principal component analysis also indicated the reliability of this result. Correlation analysis revealed that the storage stability of the rice grain quality was significantly and negatively correlated with the amylose starch content and malondialdehyde content. The average value and median value of the amylose starch content in high-storage-stability varieties are 10.7% and 6.99% lower than those in sensitive varieties, respectively. Therefore, the major feature of rice varieties with high storage stability is a low amylose starch content. This study provides valuable theoretical insights into the safe storage of rice grains and the selection and breeding of rice varieties with high storage stability.

## 1. Introduction

Rice is the world’s most important food crop, eaten by half of the world’s population [1]. Typically, rice grains undergo a storage period following harvest for the purpose of meeting consumer demand across the entire year [2]. However, rice grains generally go through a series of biochemical reactions during storage, such as respiration, enzymatic reactions, and substance metabolism [3]. These processes, along with environmental factors, can cause a deterioration in rice grain quality [4,5]. Therefore, maintaining and improving the quality stability during storage is crucial for ensuring a sustained supply of high-quality rice.

Rice grain quality is normally evaluated by considering three dimensions: the processing quality, appearance quality, and eating quality [6]. The storage stability of rice grain quality is a multifaceted trait influenced by numerous factors, such as genetic characteristics [7,8], storage methods [9], and environmental conditions [10]. Storage under nitrogen gas conditions has been found to reduce changes in the α-amylase activity, free sulfhydryl content, fatty acid value, electrical conductivity, microstructure, and pasting properties of rice grains compared to conventional storage [11,12]. Low-temperature storage is generally more effective in maintaining lower fatty acid values and stable rice grain quality than high-temperature storage conditions [13]. High temperature and high humidity during storage negatively impact some groups in the primary starch structure, resulting in changes in the internal arrangement of starch molecules and ultimately affecting the rice grain quality [14]. Furthermore, physiological and biochemical indices such as lipoxygenase activity, peroxidase activity, superoxide dismutase activity, malondialdehyde content, and α-amylase activity have been identified as key factors influencing the storage stability of rice grains [15,16]. Some studies suggest that lipoxygenase-deficient rice varieties exhibit improved storage stability, contributing to better quality retention during storage [17]. Malondialdehyde content tends to increase, and antioxidant enzyme activities decrease significantly after rice storage [18]. During rice storage, the sulfhydryl groups of proteins at the periphery of the starch in the interior of rice grains undergo oxidation to form disulfide bonds. This process causes the proteins to create a strong reticulation around the starch, which restricts the starch’s ability to swell and reduces its suppleness [19]. Pasty properties, such as peak viscosity, setback, and final viscosity, show varying changes after storage compared to fresh rice [20]. Consequently, the decline in eating quality after rice storage is primarily attributed to an increase in the hardness and chewiness of cooked rice, along with a decrease in its elasticity and adhesion.

Many studies have explored the changes in the rice grain quality during storage, focusing on different varietal types or storage conditions. However, there is limited research specifically targeting rice varieties with quality storage stability. Additionally, there is a lack of indicators that can accurately reflect this trait. In this study, 34 rice varieties widely cultivated in rice production areas were selected as experimental materials to investigate the changes in rice grain quality after natural storage for one year, analyzing the characteristics of rice at maturity. The objective is to screen rice varieties that are resistant to deterioration in rice grain quality during storage and to elucidate their storage stability characteristics. This study provides valuable insights for selecting and producing rice varieties that are resilient to quality loss during storage.

## 2. Results

### 2.1. Variable Amplitude

The storage stability of rice quality varied considerably with varieties. After storage, regarding processing quality, the brown rice rate, milled rice rate, and head milled rice rate were reduced by 1.04%, 1.34%, and 2.09%, respectively. Regarding appearance quality, the chalkiness degree of rice grains increased from 6.61 to 8.54, reflecting a variability range of 3.16% to 72.0%. On average, this value increased by 32.4%. Regarding eating quality, the appearance value of cooked rice decreased from 6.82 to 5.53, reflecting a variability range of −40.6% to −3.13% and a coefficient of variation of −52.6%. On average, this value dropped by 18.7%. The texture value of cooked rice declined from 6.47 to 5.24, exhibiting a variability range of −36.1% to −3.56% and a coefficient of variation of −45.8%. This value decreased by an average of 19.1%. The taste value of cooked rice was reduced from 65.1 to 55.9, showing a variability range of −28.8% to −0.80% and a coefficient of variation of −51.6%. The average decrease in taste score was 14.2% (Table 1).

### 2.2. Screening of Rice Varieties

#### 2.2.1. Construction of the Comprehensive Evaluation Index System

**Figure 1:**
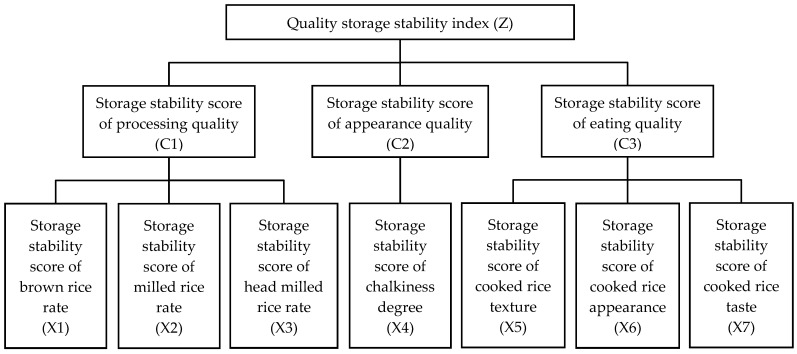
The hierarchical analytical structural model for the comprehensive evaluation of the storage stability of the rice grain quality.

In this study, hierarchical analysis was employed to evaluate the storage stability of the rice grain quality by considering three key dimensions: the storage stability score of processing quality (C1), storage stability score of appearance quality (C2), and storage stability score of eating quality (C3). Among these, processing quality was composed of three critical dimensions: the storage stability score of brown rice rate (X1), storage stability score of milled rice rate (X2), and storage stability score of head milled rice rate (X3). The storage stability score of chalkiness degree (X4) was chosen as the core indicator for the storage stability score of appearance quality. The eating quality was composed of three critical dimensions: the storage stability score of cooked rice appearance (X5), storage stability score of cooked rice texture (X6), and storage stability score of cooked rice taste (X7). A hierarchical analytical structure for the evaluation of the storage stability of rice grain quality was established, as shown in Figure 1. The judgment matrix was constructed based on related theoretical and practical experience, as listed in Table 2. The matrix underwent a consistency test, confirming its reliability (Table 2, CR < 0.10). The weighted values for the storage stability scores of the rice grain quality were calculated based on Table 2 and the methodology and formulae outlined in Section 4.5.2. The weight values of the processing quality, appearance quality, and eating quality are 0.143, 0.286, and 0.571, respectively. Furthermore, the weight values of the brown rice rate, milled rice rate, head milled rice rate, chalkiness degree, cooked rice appearance, cooked rice texture, and cooked rice taste are 0.0149, 0.0369, 0.0910, 0.286, 0.060, 0.148, and 0.364, respectively (Table 3).

#### 2.2.2. The Quality Storage Stability Index

**Figure 2:**
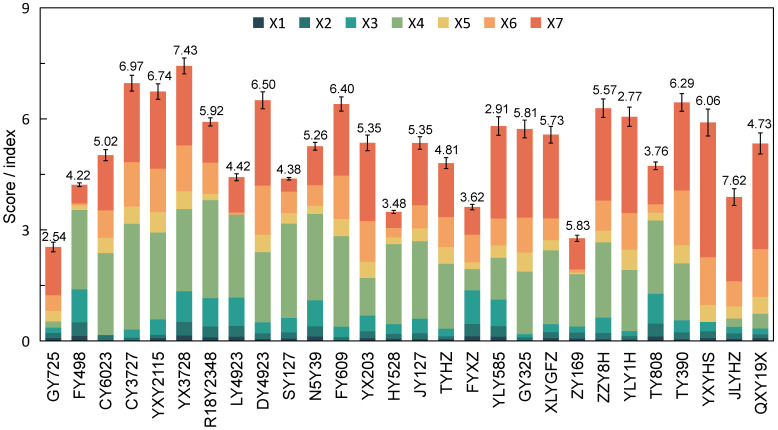
The quality storage stability index of tested cultivars. X1: storage stability score of brown rice rate; X2: storage stability score of milled rice rate; X3: storage stability score of head milled rice rate; X4: storage stability score of chalkiness degree; X5: storage stability score of cooked rice texture; X6: storage stability score of cooked rice appearance; X7: storage stability score of cooked rice taste.

In this study, the evaluation indices for the storage stability of the rice grain quality were calculated using the normalization method. An evaluation system was established with scores ranging from 0 to 10. The mean storage stability scores for the seven rice grain quality dimensions are as follows: 4.47 for X1, 4.82 for X2, 4.12 for X3, 5.75 for X4, 5.84 for X5, 5.22 for X6, and 5.21 for X7. The quality storage stability index was derived from X1, X2, X3, X4, X5, X6, and X7 and their corresponding weights (Figure 2). The higher the index, the more resistant the rice is to deterioration in rice grain quality during storage. For the rice varieties tested in this study, the mean quality storage stability index of rice grains was 5.28.

#### 2.2.3. Screening of Rice Varieties with High Storage Stability for Quality

**Figure 3:**
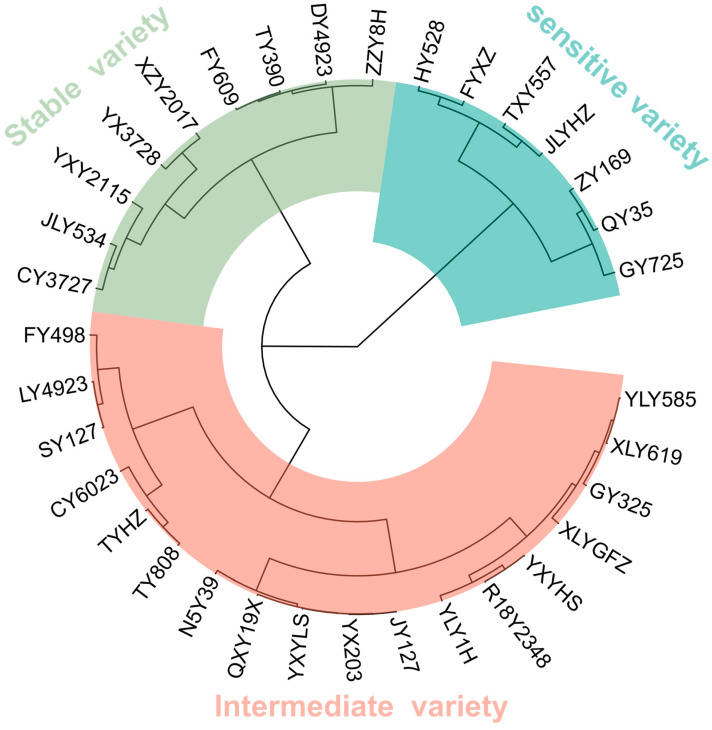
The cluster analysis of storage stability of the quality of rice.

A systematic cluster analysis method was employed to categorize the quality storage stability index of the tested varieties. Three types of varieties were identified: high storage stability of rice grain quality (a stable variety), low storage stability of rice grain quality (a sensitive variety), and intermediate storage stability of rice grain quality (an intermediate variety). From the cluster analysis, the varieties were grouped as follows: nine stable rice varieties, eighteen intermediate rice varieties, and seven sensitive rice varieties, accounting for 26.5%, 52.9%, and 20.6% of the total varieties, respectively (Figure 3).

**Figure 4:**
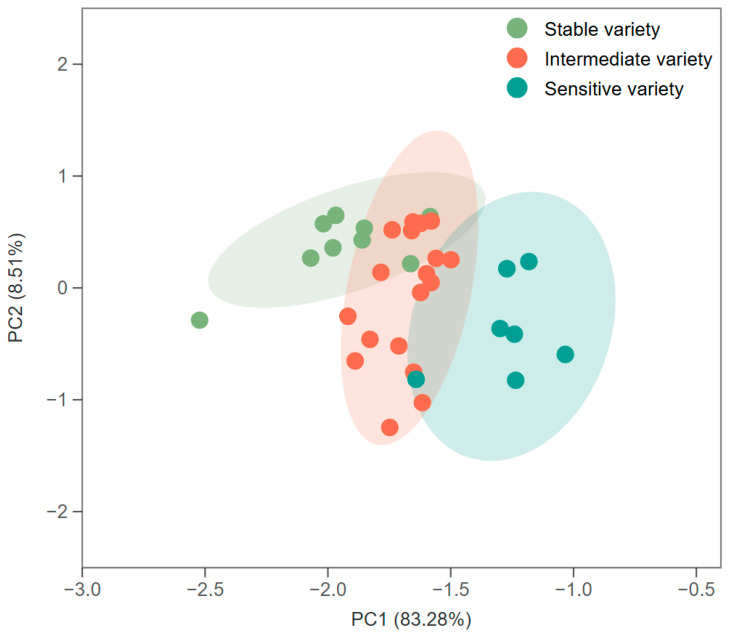
Principal component analysis of different rice varieties. Stable varieties: XZY2017, YX3728, JLY534, CY3727, YXY2115, DY4923, TY390, FY609, and ZZY8H. Intermediate varieties: YLY1H, R18Y2348, YXYHS, XLY619, YLY585, GY325, XLYGFZ, YXYLS, YX203, JY127, QXY19X, N5Y39, CY6023, TYHZ, TY808, LY4923, SY127, and FY498. Sensitive varieties: JLYHZ, TXY557, FYXZ, HY528, QY35, ZY169, and GY725.

A principal component analysis (PCA) was performed to verify the validity and applicability of the three screened categories of varieties with different storage stabilities of rice grain quality. The results of the PCA showed that these varieties could be effectively classified into three categories (Figure 4). This result is in general agreement with the results of the cluster analysis, which indicates the reliability of the results of using hierarchical analysis to classify the storage stability of rice grain quality.

### 2.3. Characteristics of Rice Varieties with High Storage Stability

**Figure 5:**
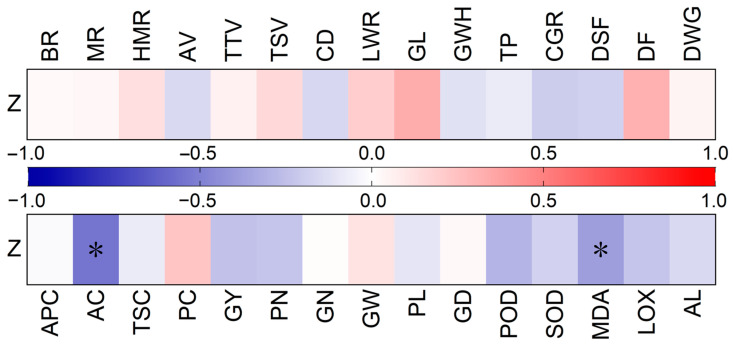
The correlation analysis (*p* < 0.05). Z: quality storage stability index; BR: brown rice rate; MR: milled rice rate; HMR: head milled rice rate; AV: appearance value of cooked rice; TTV: texture value of cooked rice; TSV: taste value of cooked rice; CD: chalkiness degree; LWR: grain length–width ratio; GL: grain length; GWH: grain width; TP: transparency; CGR: chalkiness grain rate; DSFs: days from sowing to flowering; DFs: days of filling period; DWGs: days of whole growing period; APC: amylopectin starch content; AC: amylose starch content; TSC: total starch content; PC: protein content; GY: grain yield; PN: panicle number; GN: grain number per panicle; GW: grain weight; PL: panicle length; GD: grain density; POD: peroxidase activity; SOD: superoxide dismutase activity; MDA: malondialdehyde content; LOX: lipoxygenase activity; AL: α-amylase activity. *: significant at *p* < 0.05 levels.

As depicted in Figure 5, the quality storage stability index is significantly and negatively correlated with the amylose starch content and malondialdehyde content in rice grains. However, the quality storage stability was not found to be significantly linked to the amylopectin starch content, total starch content, protein content, superoxide dismutase activity, peroxidase activity, lipoxygenase activity, and α-amylase activity. The quality storage stability index is not significantly correlated with the storage stability score of processing quality (brown rice rate, milled rice rate, and head milled rice rate), storage stability score of appearance quality (chalkiness degree), and storage stability score of eating quality (cooked rice appearance, cooked rice texture, and cooked rice taste). Meanwhile, the quality storage stability index is not significantly related to the grain size (length–width ratio, grain length, and grain width), key growth stages (the days from sowing to flowering, the filling period, and the whole growing period), rice yield and its components (panicle number, grain number per panicle, and grain weight), and panicle shape (panicle length and grain density). 

As shown in Table 4, on average, the stable variety exhibits a 3.80% and 10.7% lower amylose starch content than the intermediate variety and the sensitive variety, respectively. Meanwhile, the malondialdehyde content of the stable rice variety is 3.31% and 5.82% lower than that of the intermediate variety and the sensitive variety, respectively. From the median value, compared to sensitive varieties, stable varieties have a 6.99% lower amylose starch content while only a 1.58% lower malondialdehyde content. Therefore, the primary characteristic of rice varieties with high storage stability was identified as a low amylose starch content.

## 3. Discussion

### 3.1. Comprehensive Evaluation and Variety Screening for Quality Storage Stability in Rice Grains

As living standards continue to improve alongside economic development, consumer demand for high-quality rice has significantly increased. However, storage can cause a deterioration in the quality of rice grains [21]. Evaluating the storage stability of rice grain quality has become a crucial challenge. Rice grain quality is generally assessed through three indicators: the processing quality, appearance quality, and eating quality. Furthermore, the processing quality is composed of the brown rice rate, milled rice rate, and head milled rice rate. The eating quality is composed of the cooked rice appearance, cooked rice texture, and cooked rice taste. The evaluation of grain quality among different rice varieties cannot be effectively captured by a single index. Therefore, a more comprehensive evaluation system that accounts for multiple aspects of rice grain quality is essential.

In developing such an evaluation system, previous studies have commonly employed methods like PCA, neural networks, and other techniques [22,23]. However, the application effectiveness is inconsistent due to differences in targets and parameters. For example, PCA can eliminate the correlation effect between evaluation indicators and ensure objectivity. However, its main limitation lies in the fact that the variance contribution rate of the extracted principal components rarely reaches 100%, thus failing to capture all the information of the evaluation object [24].

Hierarchical analysis, proposed by T.L. Saaty, decomposes complex problems into a hierarchical structure and determines the relative importance of indicators through pairwise comparisons. As a multifactorial decision-making approach, it has been applied across various fields [25]. For example, Dr Zhou used hierarchical analysis to assess rice yield, quality, and nitrogen uptake and utilization under different sowing periods in the lower reaches of the Huai River Basin, identifying the temperature and light characteristics that optimize yield, quality, and efficiency in rice [26]. Dr Zhang utilized hierarchical analysis to evaluate the integrated productivity of wheat in multiple areas in Huaibei, highlighting the critical temperature and light characteristics required for achieving high integrated productivity, which is essential for the production of rice stubble wheat in various wheat areas [27].

In this study, hierarchical analysis was adopted to evaluate the quality storage stability of rice grains using seven key indicators: the brown rice rate, milled rice rate, head milled rice rate, chalkiness degree, cooked rice appearance, cooked rice texture, and cooked rice taste. And the judgment matrix was constructed (the judgment matrix also passed the consistency test) to obtain their weight values of 0.0149, 0.0369, 0.0910, 0.286, 0.060, 0.148, and 0.364, respectively. Moreover, a normalization method was employed for scoring the storage stability of the brown rice rate, storage stability of the milled rice rate, storage stability of the head milled rice rate, storage stability of the chalkiness degree, storage stability of the cooked rice appearance, storage stability of the cooked rice texture, and storage stability of the cooked rice taste. Normalization is used to eliminate scale differences by transforming data into a uniform, dimensionless scale [28]. This method is particularly useful for reducing variations in parameters between varieties subjected to different treatments by mapping the data into the (0, 1) interval [29]. The quality storage stability index of each rice variety was determined according to the individual quality storage stability scores and their weight values. The tested rice varieties were clustered into three categories, stable varieties, intermediate varieties, and sensitive varieties, based on the quality storage stability index. The validation results of the PCA also indicated the reliability of the results of using hierarchical analysis to classify the storage stability of rice grain quality.

Although using hierarchical analysis and the judgment matrix for the comprehensive evaluation of the storage stability of rice grain quality remains an emerging approach, the results effectively reflect the grain quality storage stability of rice varieties. This method represents a promising advancement in improving and refining the screening and evaluation of the storage stability of rice grain quality. Meanwhile, the identification of nine rice varieties with superior quality storage stability provides valuable guidance for rice production.

### 3.2. Characteristics of Rice Varieties with High Storage Stability

Rice grain quality is characterized by the processing, appearance, and eating quality. However, the analysis found no substantial correlation between the rice quality storage stability index and the processing quality (brown rice rate, milled rice rate, and head milled rice rate), appearance quality (chalkiness degree), and eating quality (cooked rice appearance, cooked rice texture, and cooked rice taste), as shown in Figure 5. Moreover, the rice quality storage stability index is not significantly correlated with the rice yield and its components (such as panicle number, grain number per panicle, and grain weight), panicle shape (including panicle length and grain density), or grain size (such as length–width ratio, grain length, and grain width). This indicates that the ability of rice to withstand quality degradation during storage is irrelevant to the intrinsic quality of rice grains.

The period from flowering to maturity is critical for grain filling and enrichment. It plays a vital role in the quality of rice grains [30,31]. However, as shown in Figure 5, no significant correlation is found between the rice grain quality storage stability index and the filling period, the whole growing period, and the days from sowing to flowering. This suggests that the ability of rice to maintain quality during storage is not significantly correlated with the length of the nutritive growth period or the reproductive growth period.

Starch and protein are the primary components of rice grains. Starch consists of amylose and amylopectin [32]. The storage stability of rice is significantly and negatively correlated with the amylose starch content but not significantly related to the amylopectin starch content, total starch content, and protein content (Figure 5). During storage, starch molecules are degraded by α-amylase, β-amylases, and debranching enzymes, leading to more significant changes in the fine structure of amylose starch, which directly affects the pasting characteristics of rice grains [5]. Rice varieties with a lower amylose starch content are more likely to maintain the stability of their amylose starch structure after storage. This stability of the amylose starch structure results in more stable pasting characteristics, which are essential for maintaining rice quality, especially in terms of texture and taste after cooking [33]. Table 4 shows that the mean value of the amylose starch content of the stable variety is 10.7% lower than that of the sensitive variety. In terms of the median value, it is 6.99% lower than that of the sensitive variety. This demonstrates that the characteristics of rice varieties with a high storage stability of grain quality include a low amylose starch content.

Superoxide dismutase activity, peroxidase activity, malondialdehyde content, lipoxygenase activity, and α-amylase activity have been identified as factors profoundly influencing the storage stability of rice [34,35]. In this study, the grain quality storage stability was found to be significantly and negatively correlated with malondialdehyde content but not significantly linked to peroxidase activity, superoxide dismutase activity, lipoxygenase activity, and α-amylase activity (Figure 5). Malondialdehyde is a product of lipid peroxidation and is commonly used as a biomarker for oxidative stress and cellular damage [36]. During storage, lipids in rice grains undergo peroxidation due to environmental factors, such as oxygen, temperature, and humidity [37]. This process is a free radical chain reaction: unsaturated fatty acids are attacked by active oxygen species (superoxide anions and hydroxyl radicals), forming lipid radicals. Then, these lipid radicals react with oxygen to generate lipid peroxides, which decompose to form malondialdehyde [16,38]. A low malondialdehyde content in rice grains implies relatively low lipid peroxidation, suggesting that the fats in rice grains are well preserved and not excessively subjected to oxidative damage, and lipids are not susceptible to oxidative reactions. Lipid oxidation produces undesirable compounds that deteriorate the quality of rice [39]. Therefore, maintaining a low malondialdehyde content in rice during storage contributes to the preservation of its quality. Table 4 shows that the malondialdehyde content of the stable rice variety is 5.82% lower than that of the sensitive variety. Therefore, to some extent, a low malondialdehyde content can also reflect the high storage stability of rice quality.

This study makes several recommendations on rice storage. First, regarding temperature and humidity control, it is recommended that rice grain storage warehouses be equipped with effective temperature and humidity regulating equipment (air conditioners, dehumidifiers, etc.) to control the warehouse temperature at about 10 °C and keep the relative humidity at about 65%, in order to delay the deterioration of the rice grain quality. Under high-temperature and high-humidity conditions, the quality of rice grains declines due to a significant increase in the content of free fatty acids and peroxides and a rise in the content of volatile compounds such as aldehydes, ketones, and furans. Second, this study promotes gas-conditioned storage technology. It is recommended to use nitrogen or carbon dioxide to replace the air and reduce the oxygen content to less than 5%, which can effectively inhibit rice respiration and prolong the storage period of rice quality. Thirdly, categorized storage methods for different varieties should be adopted. This study concluded that a low amylose starch content is a key characteristic of rice varieties with high quality storage stability. Understanding these characteristics can help industry stakeholders select rice varieties suitable for long-term storage and optimize storage conditions according to the characteristics of different varieties.

## 4. Materials and Methods

### 4.1. Trial Material

Thirty-four extensively planted rice varieties were selected as the test materials: GY725, FY498, CY6203, CY3727, YXY2115, YX3728, R18Y2348, QXY19X, LY4923, DY4923, SY127, N5Y39, FY609, YX203, HY528, TXY557, JY127, TYHZ, ZZY8H, YLY585, XLY619, XLYGFZ, YXYLS, ZY169, YLY1H, XZY2017, GY325, TY808, TY390, QY35, YXYHS, FYXZ, JLYHZ, and JLY534.

### 4.2. Trial Site and Meteorological Information

**Figure 6:**
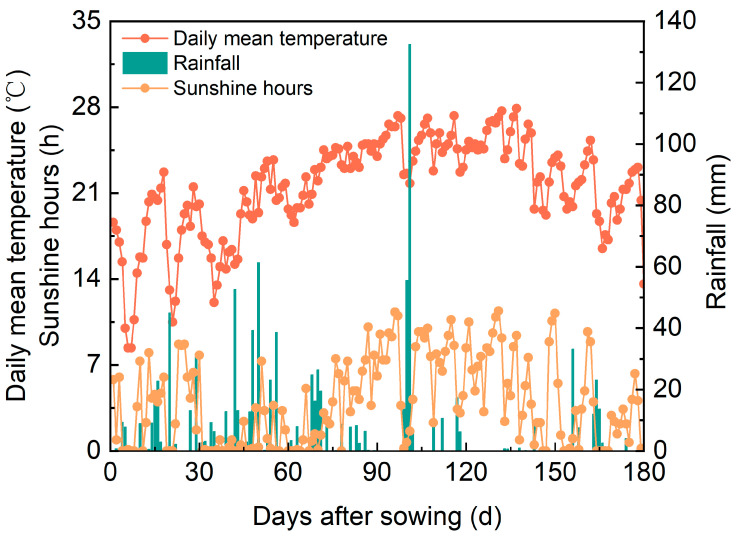
Daily mean temperature, sunshine hours, and rainfall during rice growth season in 2022.

The trial was performed in 2022 at the farm at Guizhou Rice Research Institute (altitude: 1150 m, latitude: 26°41′ N, and longitude: 106°66′ E). The previous crop was a winter fallow field, and the soil was classified as yellow loam. The physical and chemical properties of the soil in the tillage layer were 13.9 g/kg of organic matter, 1.20 g/kg of total nitrogen, 86.7 mg/kg of alkaline dissolved nitrogen, 32.8 mg/kg of available phosphorus, 87.7 mg/kg of available potassium, and a pH of 6.22. The daily mean temperature, sunshine hours, and rainfall of the rice planting season are shown in Figure 6.

### 4.3. Trial Design

The experimental rice varieties were sown on 11 April and transplanted on 25 May with a planting spacing of 20 cm × 30 cm. The chemical fertilizers urea, phosphate, and potassium were applied to all plots. Urea (150 kg/ha) was applied in three splits (4:3:3; pre sowing, 4-leaf stage, and panicle initiation). Meanwhile, P fertilizer (P_2_O_5_) was applied at the rate of 75 kg/ha pre sowing, while K fertilizer (K_2_O) was applied at the rate of 150 kg/ha in two splits, one pre sowing and the other at panicle initiation. Pest and weed management were implemented according to local conventional practices for high-yielding cultivation.

### 4.4. Sampling and Measurement

#### 4.4.1. Rice Grain Quality

The processing quality (brown rice rate, milled rice rate, and head milled rice rate) of rice grains before storage (fresh rice) and after one year of storage (stored rice) was evaluated following the determination method of the National Standard of the People’s Republic of China (GB/T17891-2017, High-Quality Rice) [32]. The values for the appearance quality (chalkiness degree) of fresh rice grains and stored rice grains were calculated using a rice appearance quality detector (Hangzhou Wanshen Detection Technology Co., Ltd., Hangzhou, China). The eating quality (appearance, texture, and taste of cooked rice) of fresh rice grains and stored rice grains was evaluated using a rice taste analyzer (Satake Column Co., Higashi-Hiroshima, Japan) [6].

#### 4.4.2. Grain Yield and Its Composition

The panicle number was counted by surveying 100 holes within each plot at maturity. The grain number per panicle, grain weight, and panicle length were measured from 10 holes in each plot according to the average tiller. The grain yield was obtained by weighing 100 holes harvested per plot at maturity and converted to a standard moisture content of 13.5%. Grain density = grain number per panicle/panicle length [40].

#### 4.4.3. Key Growth Stages of Rice

The dates of sowing, flowering, and maturity for each rice variety were recorded in detail. For the date of sowing, the date when the seeds of each rice variety were sown in the experimental field was precisely recorded. For the date of flowering, we defined it as the time when 50% of the rice plants in the field flowered. To accurately record this period, we made several observations at different times of the day, especially when the rice was approaching the flowering stage. For the date of maturity, this was determined as the date when 80% of the rice grains in the field turned yellow. We continuously monitored grain color changes to ensure that we accurately determined the date (Supplementary Table S1).

#### 4.4.4. Grain Size and Visual Characteristics

The grain length–width ratio, grain length, grain width, transparency, and chalkiness grain rate were determined using a rice appearance quality tester (Wanshen Testing Technology Co., Ltd., Hangzhou, China). Before using the rice appearance quality tester, a calibration of the instrument was carried out according to the manufacturer’s instructions. In total, 600–800 rice grains were randomly selected from each rice variety, and these grains were spread evenly on the sample tray of the tester. Each measurement was repeated three times to ensure the accuracy and reliability of the data.

#### 4.4.5. Major Component Content in Grains

According to the national standard of the People’s Republic of China (GB/T17891-2017, High-Quality Rice Grain), rice grains were ground into a fine powder using a high-speed mill, and then the protein content, amylose content, amylopectin content, and total starch content of grains were quantitatively determined. For the determination of protein content, the Kjeldahl method was used. The amylose and amylopectin contents were determined by a dual-wavelength method. The sum of the amylose content and amylopectin content is the total starch content. Each determination was repeated three times.

#### 4.4.6. Physiological Indicator

At maturity, 200–300 grains were randomly selected and placed in liquid nitrogen for one minute. Then, the grains were stored in an ultra-low-temperature freezer for further analysis of physiological indicators. The peroxidase activity was determined using the guaiacol method [41]. The superoxide dismutase activity was measured using the nitrogen blue tetrazolium photochemical reduction method [42]. The malondialdehyde content was quantified by the thiobarbituric acid method [43]. The lipoxygenase activity was determined using the fluorescence method [44]. The α-amylase activity was assessed by the 3,5-dinitrosalicylic acid method [45].

### 4.5. Formula Calculation and Statistical Analysis

#### 4.5.1. Variable Amplitude of the Rice Grain Quality 

The variable amplitude of the processing quality (appearance or eating quality) of rice grain = [processing quality (appearance or eating quality) of stored rice grain − processing quality (appearance or eating quality) of fresh rice grain]/processing quality (appearance or eating quality) of fresh rice grain × 100%.

#### 4.5.2. Comprehensive Evaluation

The storage stability of the rice grain quality was evaluated based on the method provided by Liu et al. [22] and Zhou et al. [26]. The steps were as follows:

(1) Storage stability score calculation (normalization method).

The storage stability score of the rice grain quality (the processing, appearance, and eating quality) was calculated using the normalization method on a scale of 0–10 points. Based on the principle that a larger value indicates better performance, the formula for the storage stability score of the processing quality and eating quality is as below:

(1) $$r_{i}\;=\frac{x_{i}\;-\;\min x_{i}}{\max x_{i}\;\;-minx_{i}}\times 10$$

The formula for the storage stability score of the appearance quality is as below:

(2) $$r_{i}\;=\frac{\max x_{i}{\;-x}_{i}}{\max x_{i}\;-minx_{i}}\times 10$$

where $r_{i}\;$ denotes the storage stability score of the rice grain quality, $x_{i}$ is the variable amplitude of rice grain quality, and $m\mathrm{ax}x_{i}$ and $\min x_{i}$ represent the maximum and minimum values of each evaluation index, respectively.

(2) Weighted value of the storage stability score of the rice grain quality determined by the hierarchical analysis method.

The hierarchical analysis method involves constructing a hierarchical structural model to analyze the factors and their relationships in a complex system, structuring and hierarchizing the problem. Then, the elements of each level are compared two by two. According to a certain scale theory, the relative importance of the comparison scale is obtained, forming a judgment matrix. The maximum eigenvalue of the judgment matrix and its eigenvector are calculated to obtain the order of importance of the elements in each level to an element in the upper level, establishing a weight vector. The specific steps are as follows:

I. Establishing a recursive hierarchical structure.

The hierarchical analysis structural model was established based on the interrelationships and affiliations among the indicators of the rice grain quality. The ultimate goal of the evaluation is located at the top of the model, and specific evaluation indicators are located at the lowest level of the model.

II. Constructing a judgment matrix.

The judgments are given in numerical form based on the relative importance of each factor at each level (refer to Table 5 for specific guidelines). Based on this, a judgment matrix (A) is constructed as follows:

(3) $$A=\;{\left(b_{ij}\right)}_{n\times n},(i,j=1,2,\cdots n)$$

III. Calculating the weighted value.

The columns of the judgment matrix are normalized:

(4) $${\bar{b}}_{ij}\;=b_{ij}/\sum_{k=1}^{n}b_{kj}\;\;\;\;(i,j=1,2,\cdots ,n)$$

The sum (${\bar{w}}_{i}$) of the data in each row of the normalized judgment matrix is computed:

(5) $${\bar{w}}_{i}\;=\sum_{j=1}^{n}{\bar{b}}_{ij}\;\;\;\;\;(i=1,2,\cdots ,n)$$

The feature vector ($w_{i}$) is obtained by performing a normalization on ${\bar{w}}_{i}$:

(6) $$w_{i}\;={\bar{w}}_{i}/\sum_{i=1}^{n}{\bar{w}}_{i}\;\;\;\;\;\;(i=1,2,\cdots ,n)$$

The eigenvector ($Aw_{i}$) is calculated using the following formula:

(7) $$Aw_{i}\;=A\times w_{i}$$

The weight value ($W_{i}$) of the hierarchical element to its subordinate element is calculated by normalizing the eigenvector ($Aw_{i}$).

IV. The consistency test.

The largest eigenroot ($\lambda_{max}$) of the judgment matrix is obtained from the following formula:

(8) $$\lambda_{max}\;=\frac{Aw_{i}}{w_{i}}$$

The consistency index (*CI*) is calculated according to the following formula:

(9) $$CI=\frac{\lambda_{max}-n}{n-1}$$

The consistency ratio (CR) is derived from the following formula:

CR = *CI*/RI (10)

where RI is an average stochastic consistency index, and its value is detailed in Table 6. A CR value < 0.10 indicates that this judgment matrix has satisfactory consistency. Otherwise, the judgment matrix needs to be adjusted.

(3) Calculating the quality storage stability index.

The quality storage stability index (Z) is obtained by multiplying each storage stability score of the rice grain quality ($r_{i}$) with the corresponding weight value ($W_{i}$), expressed as the following formula:

(11) $$Z\;\;=\;\sum_{i=1}^{n}W_{i}r_{i}$$

#### 4.5.3. Statistical Analysis

The data were analyzed using Excel 2019 and SPSS 22.0 statistical software. Graphing was conducted using Origin 2021 and the R programming language (R version 4.4.2). Significant differences between sample means were analyzed using the least significant difference (LSD) test. The PCA group analysis of each variety group was based on the varieties screened by cluster analysis.

## 5. Conclusions

This study evaluated the changes in the grain quality of 34 rice varieties widely grown in rice production regions after one year of natural storage. The characteristics of rice at maturity were analyzed. The chalkiness degree increased significantly by 32.4%. The cooked rice appearance, texture, and taste quality decreased significantly by 18.7%, 19.1%, and 14.2%, respectively. In the comprehensive evaluation index system of the rice grain quality storage stability, the weight value of the brown rice rate, milled rice rate, head milled rice rate, chalkiness degree, cooked rice appearance, cooked rice texture, and cooked rice taste is 0.0149, 0.0369, 0.0910, 0.286, 0.060, 0.148, and 0.364, respectively. Nine rice varieties were identified as having high storage stability using the cluster analysis and normalization method. Correlation analysis revealed that the storage stability of the rice grain quality was significantly and negatively correlated with the amylose starch content and malondialdehyde content. Notably, the amylose starch content of the high-storage-stability varieties was significantly lower than that of the sensitive varieties, with the mean and median values reduced by 10.7% and 6.99%, respectively. The primary characteristic of rice varieties with high storage stability was identified as a low amylose starch content. This study provides valuable theoretical support for the safe storage of rice grains and the selection and breeding of rice varieties with high storage stability.

## Figures and Tables

**Table 1:** The variable amplitude of the grain quality after rice storage.

Index			Range	Mean Value	CV (%)
		Brown rice rate (%)	78.1–81.6	79.9	0.98
	The processing quality	Milled rice rate (%)	63.1–74.9	70.2	3.49
		Head milled rice rate (%)	51.8–71.0	61.9	6.75
The fresh rice	The appearance quality	Chalkiness degree (%)	1.25–11.9	6.61	36.0
		Appearance values of cooked rice	5.70–7.75	6.82	6.98
	The eating quality	Texture values of cooked rice	5.20–7.35	6.47	7.88
		Taste values of cooked rice	55.5–74.3	65.1	6.66
		Brown rice rate (%)	76.2–81.0	79.1	1.63
	The processing quality	Milled rice rate (%)	64.2–73.7	69.3	3.36
		Head milled rice rate (%)	53.6–70.0	60.6	6.03
The stored rice	The appearance quality	Chalkiness degree (%)	2.15–20.0	8.54	37.1
		Appearance values of cooked rice	3.70–6.55	5.53	12.3
	The eating quality	Texture values of cooked rice	3.55–6.20	5.24	13.5
		Taste values of cooked rice	43.1–67.2	55.9	10.9

**Table 2:** The judgment matrix and consistency test.

Judgment Matrix and Consistency Test				
Judgment matrix	Z	C1	C2	C3
	C1	1	1/2	1/4
	C2	2	1	1/2
	C3	4	2	1
Consistency test	$\lambda_{max}\;$= 3.00, *CI* = 0.00, RI = 0.00, CR = 0.000 < 0.10			
Judgment matrix	C1	X1	X2	X3
	X1	1	1/3	1/5
	X2	3	1	1/3
	X3	5	3	1
Consistency test	$\lambda_{max}\;$= 3.04, *CI* = 0.019, RI = 0.58, CR = 0.033 < 0.10			
Judgment matrix	C3	X5	X6	X7
	X5	1	1/3	1/5
	X6	3	1	1/3
	X7	5	3	1
Consistency test	$\lambda_{max}\;$= 3.04, *CI* = 0.019, RI = 0.58, CR = 0.033 < 0.10			

Z: quality storage stability index; C1: storage stability score of processing quality; C2: storage stability score of appearance quality; C3: storage stability score of eating quality; X1: storage stability score of brown rice rate; X2: storage stability score of milled rice rate; X3: storage stability score of head milled rice rate; X5: storage stability score of cooked rice texture; X6: storage stability score of cooked rice appearance; X7: storage stability score of cooked rice taste.

**Table 3:** Weight values of comprehensive evaluation indicators for the rice quality storage stability.

Top Level Z	Quality Storage Stability Index (Z)						
Level C versus top level Z	(C1)			(C2)	(C3)		
	0.143			0.286	0.571		
Level X versus top level Z	(X1)	(X2)	(X3)	(X4)	(X5)	(X6)	(X7)
	0.0149	0.0369	0.0910	0.286	0.060	0.148	0.364

C1: storage stability score of processing quality; C2: storage stability score of appearance quality; C3: storage stability score of eating quality; X1: storage stability score of brown rice rate; X2: storage stability score of milled rice rate; X3: storage stability score of head milled rice rate; X4: storage stability score of chalkiness degree; X5: storage stability score of cooked rice texture; X6: storage stability score of cooked rice appearance; X7: storage stability score of cooked rice taste.

**Table 4:** The key characteristics of rice varieties with high storage stability.

Variety Type	Index	Mean Value	Median Value	Variable Amplitude (%)
Stable variety	Amylose starch content (%)	17.4	17.7	15.4–19.8
	Malondialdehyde content (%)	3.24	3.33	2.49–3.55
Intermediate variety	Amylose starch content (%)	18.1	17.8	15.3–22.6
	Malondialdehyde content (%)	3.35	3.35	2.90–3.66
Sensitive variety	Amylose starch content (%)	19.5	18.9	16.8–22.9
	Malondialdehyde content (%)	3.44	3.39	3.27–3.62

**Table 5:** The scales and their meaning.

Scale	Meaning
1	Equal importance of both indicators
3	Slightly more important for one indicator compared to the other
5	Noticeably more important for one indicator compared to the other
7	Extremely more important for one indicator compared to the other
9	Vitally more important for one indicator compared to the other
2, 4, 6, 8	The median of two adjacent judgments

**Table 6:** The RI value.

Order *n*	1	2	3	4	5	6	7	8	9
RI value	0.00	0.00	0.58	0.90	1.12	1.24	1.32	1.41	1.45

## Data Availability

All data generated or analyzed during this study are included in this published article. The data used to support the findings of this study can be made available by the corresponding author upon request.

## Supplementary Materials

The following supporting information can be downloaded at: https://www.mdpi.com/article/10.3390/plants14030356/s1, Table S1: Key growth stages for rice variety.
