# Supplementary material for: Evaluation of Rice Quality Storage Stability: From Variety Screening to Trait Identification
Source: Plants (Basel). 2025 Jan 24;14(3):356. doi: 10.3390/plants14030356 (PMC11820699; doi:10.3390/plants14030356)
Supplement: Supplementary file 1 [file plants-14-00356-s001.zip › plants-3369069-supplementary.pdf]

Supplemental Table S1 Key growth stages for rice variety

Variety name	Dates of sowing	Dates of flowering	Dates of maturity
GY725	April 11	August 10	September 16
FY498	April 11	August 8	September 20
CY6023	April 11	August 8	September 28
CY3727	April 11	August 14	September 26
YXY2115	April 11	August 8	September 27
YX3728	April 11	August 10	September 22
R18Y2348	April 11	August 10	September 30
LY4923	April 11	August 13	September 28
DY4923	April 11	August 9	October 1
SY127	April 11	August 13	September 23
N5Y39	April 11	August 8	September 26
FY609	April 11	August 6	September 18
YX203	April 11	August 9	September 26
HY528	April 11	August 14	October 1
JY127	April 11	August 10	September 27
TYHZ	April 11	August 9	September 21
FYXZ	April 11	August 15	September 27
QY35	April 11	August 9	September 22
YLY585	April 11	August 10	September 29
GY325	April 11	August 4	September 19
XLY619	April 11	July 30	September 19
XLYGFZ	April 11	July 30	September 22
ZY169	April 11	August 8	September 23
TXY557	April 11	August 5	September 20
ZZY8H	April 11	August 14	September 30
YLY1H	April 11	August 16	October 1
XZY2017	April 11	July 30	September 16
TY808	April 11	August 9	September 27
TY390	April 11	July 30	September 16
YXYLS	April 11	August 6	September 20
YXYHS	April 11	August 7	September 21
JLYHZ	April 11	August 14	October 1
JLY534	April 11	August 17	October 1
QXY19X	April 11	August 14	September 27
